# Di-μ-chlorido-bis­{[2-({[2-(2-pyrid­yl)eth­yl](2-pyridylmeth­yl)amino}meth­yl)phenol]zinc(II)} bis­(perchlorate) dihydrate

**DOI:** 10.1107/S1600536810003259

**Published:** 2010-01-30

**Authors:** Sara E. Coelho, Geovana G. Terra, Adailton J. Bortoluzzi

**Affiliations:** a Depto. de Química, Universidade Federal de Santa Catarina, 88040-900 Florianópolis, SC, Brazil

## Abstract

The title compound, [Zn_2_Cl_2_(C_20_H_21_N_3_O)_2_](ClO_4_)_2_·2H_2_O, consists of a dinuclear Zn^II^ cationic complex, two disordered perchlorate anions and two water mol­ecules as solvate. The [Zn_2_(μ-Cl)_2_(H*L*)_2_]^2+ ^cation [H*L* is 2-({[2-(2-pyrid­yl)eth­yl](2-pyridylmeth­yl)amino}meth­yl)phenol] has a centrosymmetric structure with the Zn^II^ ions in a distorted octa­hedral environment surrounded by an N_3_OCl_2_ donor set. H*L* acts as a tetra­dentate ligand through three N atoms from one amine group and two pyridyl arms and one O atom from the phenolic arm. The three N-donor sites of the H*L* ligand are arranged in meridional fashion, with the pyridine rings coordinated in *trans* positions with respect to each other. Consequently, the amine and phenol groups are *trans* to the asymmetric di-μ-chlorido exogenous bridges. A polymeric chain is formed along [010] by *C*(12)*R*_4_^2^(8) inter­molecular hydrogen bonding. The perchlorate anion is disordered and was modelled by two sites in a 0.345 (18):0.655 (18) ratio. Water–perchlorate O—H⋯O inter­actions form cyclic structures, while phenol–water O—H⋯O inter­actions generate an infinite chain. In addition, weak inter­molecular C—H⋯π(Ph) inter­actions between pyridine donor and phenol acceptor groups of neighboring cations build a two-dimensional polymeric structure parallel to (110).

## Related literature

For general background to zinc enzymes, see: Parkin (2004 ▶) and for general background to mimetic models of zinc enzymes, see: Boseggia *et al.* (2004 ▶); Mancin & Tecillia (2007 ▶); Mitić *et al.* (2006 ▶); Morrow & Iranzo (2004 ▶); Rajski & Williams (1998 ▶). For the biological activity of zinc complexes, see: Beraldo & Gambino (2004 ▶); Singla & Wadhwa (1995 ▶); Zhou *et al.* (2003 ▶). For related structures, see: Ojida *et al.* (2006 ▶); Trösch & Vahrenkamp (1998 ▶); Gross & Vahrenkamp (2005 ▶). For the preparation of the H*L* ligand, see: Yan & Que (1988 ▶).
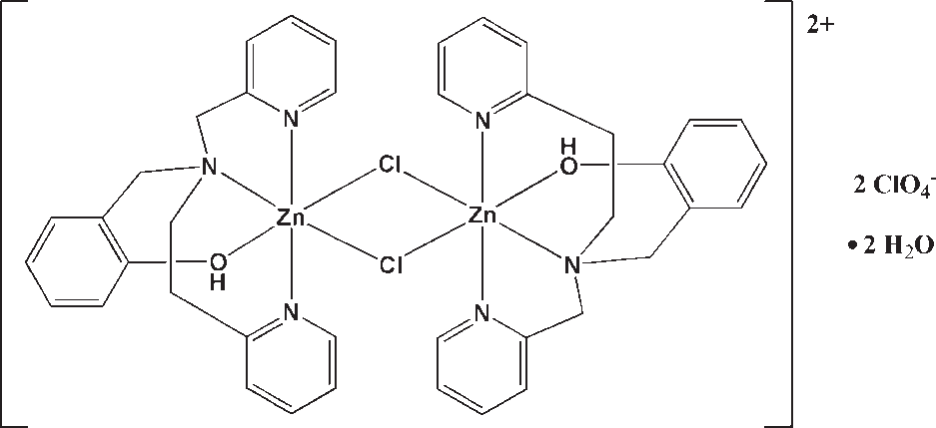


## Experimental

### 

#### Crystal data

[Zn_2_Cl_2_(C_20_H_21_N_3_O)_2_](ClO_4_)_2_·2H_2_O
*M* *_r_* = 1075.37
Monoclinic, 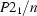

*a* = 12.3394 (14) Å
*b* = 13.2714 (9) Å
*c* = 14.751 (2) Å
β = 107.779 (9)°
*V* = 2300.2 (5) Å^3^
*Z* = 2
Mo *K*α radiation
μ = 1.34 mm^−1^
*T* = 293 K
0.50 × 0.46 × 0.33 mm

#### Data collection

Enraf–Nonius CAD-4 diffractometer
Absorption correction: ψ scan [*PLATON* (Spek, 2009 ▶) and North *et al.* (1968 ▶)] *T*_min_ = 0.554, *T*_max_ = 0.666
4253 measured reflections
4088 independent reflections
2810 reflections with *I* > 2σ(*I*)
*R*_int_ = 0.022
3 standard reflections every 200 reflections  intensity decay: 1%

#### Refinement

*R*[*F* ^2^ > 2σ(*F* ^2^)] = 0.042
*wR*(*F* ^2^) = 0.121
*S* = 1.05
4088 reflections
326 parameters
124 restraints
H-atom parameters constrained
Δρ_max_ = 0.47 e Å^−3^
Δρ_min_ = −0.38 e Å^−3^

### 

Data collection: *CAD-4 Software* (Enraf–Nonius, 1989 ▶); cell refinement: *SET4* in *CAD-4 Software*; data reduction: *HELENA* (Spek, 1996 ▶); program(s) used to solve structure: *SIR97* (Altomare *et al.*, 1999 ▶); program(s) used to refine structure: *SHELXL97* (Sheldrick, 2008 ▶); molecular graphics: *PLATON* (Spek, 2009 ▶) and *Mercury* (Macrae *et al.*, 2006 ▶); software used to prepare material for publication: *SHELXL97*.

## Supplementary Material

Crystal structure: contains datablocks global, I. DOI: 10.1107/S1600536810003259/fj2277sup1.cif

Structure factors: contains datablocks I. DOI: 10.1107/S1600536810003259/fj2277Isup2.hkl

Additional supplementary materials:  crystallographic information; 3D view; checkCIF report

## Figures and Tables

**Table 1:** Hydrogen-bond geometry (Å, °) *Cg* is the centroid of the C11–C16 ring.

*D*—H⋯*A*	*D*—H	H⋯*A*	*D*⋯*A*	*D*—H⋯*A*
O10—H10⋯O1*W*	0.93	1.67	2.598 (4)	170
O1*W*—H1*WA*⋯O2*P*	0.86	2.34	2.928 (15)	126
O1*W*—H1*WB*⋯O2*P*^i^	0.84	2.32	2.777 (15)	114
C35—H35⋯*Cg*^ii^	0.93	3.14	3.944	148

Symmetry code: (i) 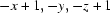; (ii) 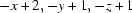.

**Fig. 1.**
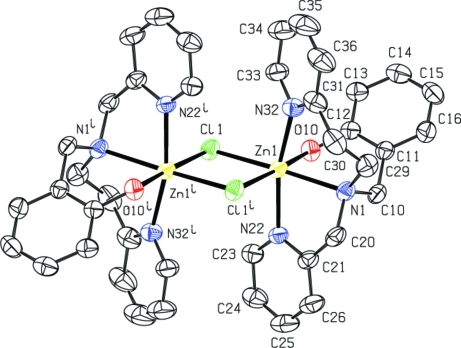
The molecular structure of (I) with partial labeling scheme. Hydrogen atoms were omitted for clarity. Displacement ellipsoids are shown at the 30% probability level. Symmetry code: -x+1, -y+1, -z+1.

**Fig. 2.**
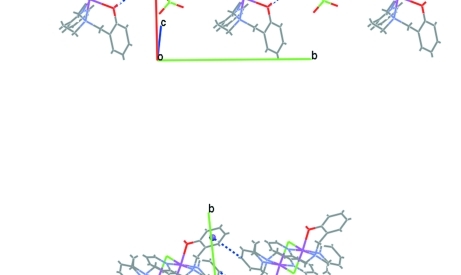
Partial packing of the title compound showing one-dimensional chain along [010] (symmetry code: -x+1, -y, -z+1) (top) and two-dimensional polymer parallel to (110) plane (symmetry code: -x+2, -y+1, -z+1) (bottom).

## supplementary crystallographic information

### Comment

Zinc is found in an all known forms of life as a trace element. It plays important roles in biological systems and its divalent ion is present in the active sites of several classes of metalloenzymes (Parkin, 2004). The design of artificial nucleases has recently received considerable interest due to their potential applications in catalysis, molecular biology and drug development (Mitić *et al.*, 2006; Morrow & Iranzo, 2004; Rajski & Williams, 1998). Intense efforts have been devoted to the improvement of mimetic models for nucleases and peptidases through mono and polynuclear complexes (Boseggia *et al.*, 2004; Mancin & Tecillia, 2007; Mitić *et al.*, 2006). Recent studies have demonstrated that zinc complexes show anti-inflammatory and anti-tumoral activity (Beraldo & Gambino, 2004; Singla & Wadhwa, 1995; Zhou *et al.*, 2003).

The cation of (I) is a dinuclear Zn^II^ complex showing centrosymmentric molecular structure with local symmetry C_i_. Zinc(II) ions are in distorted octahedral environment surrounded by N_3_O donor set of the H*L* ligand (H*L* is (2-pyridylethyl)(2-pyridylmethyl)(2-hydroxybenzyl)amine) and two Cl^-^ as exogenous bridges (Fig. 1). H*L* ligand acts as a typical four-chelating ligand and it is coordinated to the metal center through its three nitrogen atoms of the amine group and two pyridinic arms and one oxygen atom from protonated phenolic arm. The three N-donor sites of the H*L* ligand are arranged in meridional fashion, where pyridine rings are coordinated in trans positions with respect to each other. Consequently, the amine and phenol groups are trans to the asymmetric bis(µ-chloro) bridges.

Bond lengths around metal center are in the expected range and comparable to other zinc(II) complexes with N_2_O donor set (Ojida *et al.*, 2006; Trösch & Vahrenkamp, 1998; Gross & Vahrenkamp, 2005). The long distance Zn—O_phenol_ is typical for the coordination of protonated phenol group. The asymmetric bridge is due to the fact that two different groups with different trans effect are in trans positions to the chloro bridge. The distance Zn—Cl is shorter when amine group is trans to the bridge, whereas this distance is longer when phenol group is trans to µ-Cl. The cis angles are ranging from 77.69 (14)° to 100.55 (9)°, being the more closed angle restricted by a five-membered chelate ring formed by 2-methylpyridine arm. Although 2-ethylpyridine arm makes six-membered chelate ring, 2-methylpyridine arm also induces the greatest deviation from the ideal trans angle for N22—Zn1—N32.

The three-dimensional packing of (I) is governed by an extensive and interesting hydrogen bonding network (Fig. 2). Water molecules of crystallization and perchlorate anions form a cyclic structures by O1W—H···O interactions with a graph set of R_4_^2^(8). These rings link the dinuclear cations through O10—H···O1W interactions between phenol and water groups building infinite one-dimensional chains along [010] direction with C(12) graph set. In addition, weak C—H···π(phenol) intermolecular interactions between pyridine (donor) and phenol (acceptor) groups of neighboring molecules also contribute to the stabilization of the crystalline structure aggregating the linear chains in two-dimensional polymer parallel to (110) plane. The calculated distance H35···centroid_phenol_ is 3.136 Å and the angle C35—H35—Centroid is 147.55°. Finally, the molecules of (I) are stacked viewing along [100] in perpendicular projection of the linear chains.

### Experimental

H*L* ligand has been prepared according to the procedure described by Yan & Que (1988). To a solution of H*L* (0.239 g, 0.78 mmol) in methanol (10 ml) was dropped 10 ml of the suspension containing 0.291 g of Zn(ClO)_4_.6H_2_O (0.78 mmol) in methanol. The mixture was stirred for 15 minutes at room temperature. After one hour on standing, a white precipitate was formed and it was filtered off and dried under vacuum (yield 0.63 g, 74%). The white powder was recrystallized in ethanol affording colorless crystals.

### Refinement

H atoms were placed at their idealized positions with distances of 0.97 and 0.93 Å for CH_2_ and CH_Ar_, respectively. U_iso_ of the H atoms were fixed at 1.2 times of the U_eq_ of the preceding atom. Hydrogen atoms of the phenol and water of crystallization were found from Fourier difference map and treated with riding model and its U_iso_ were also fixed at 1.2 times U_eq_ of the parent O atom. Perchlorate anion is disordered with two alternative positions for all oxygen atoms. The occupancy for disordered atoms of 0.655 (18) and 0.345 (18) were refined. Although disordered O atoms and some carbon atoms show abnormal adp, all non-hydrogen atoms were refined anisotropically with positive definite thermal tensor. Further, the final indices wR_2_ decreased more than 33% (from about 0.18 to 0.1211) after anisotropic refinement of the disordered atoms.

### Crystal data

**Table:**

[Zn_2_Cl_2_(C_20_H_21_N_3_O)_2_](ClO_4_)_2_·2H_2_O	*F*(000) = 1104
*M**_r_* = 1075.37	*D*_x_ = 1.553 Mg m^−^^3^
Monoclinic, *P*2_1_/*n*	Mo *K*α radiation, λ = 0.71073 Å
Hall symbol: -P 2yn	Cell parameters from 25 reflections
*a* = 12.3394 (14) Å	θ = 8.3–17.1°
*b* = 13.2714 (9) Å	µ = 1.34 mm^−^^1^
*c* = 14.751 (2) Å	*T* = 293 K
β = 107.779 (9)°	Irregular block, colorless
*V* = 2300.2 (5) Å^3^	0.50 × 0.46 × 0.33 mm
*Z* = 2	

### Data collection

**Table:**

Enraf–Nonius CAD-4 diffractometer	2810 reflections with *I* > 2σ(*I*)
Radiation source: fine-focus sealed tube	*R*_int_ = 0.022
graphite	θ_max_ = 25.1°, θ_min_ = 1.9°
ω–2θ scans	*h* = −14→14
Absorption correction: ψ scan [*PLATON* (Spek, 2009) and North *et al.* (1968)]	*k* = −15→0
*T*_min_ = 0.554, *T*_max_ = 0.666	*l* = −17→0
4253 measured reflections	3 standard reflections every 200 reflections
4088 independent reflections	intensity decay: 1%

### Refinement

**Table:**

Refinement on *F*^2^	Primary atom site location: structure-invariant direct methods
Least-squares matrix: full	Secondary atom site location: difference Fourier map
*R*[*F*^2^ > 2σ(*F*^2^)] = 0.042	Hydrogen site location: inferred from neighbouring sites
*wR*(*F*^2^) = 0.121	H-atom parameters constrained
*S* = 1.05	*w* = 1/[σ^2^(*F*_o_^2^) + (0.0568*P*)^2^ + 1.725*P*] where *P* = (*F*_o_^2^ + 2*F*_c_^2^)/3
4088 reflections	(Δ/σ)_max_ = 0.001
326 parameters	Δρ_max_ = 0.47 e Å^−^^3^
124 restraints	Δρ_min_ = −0.38 e Å^−^^3^

### Fractional atomic coordinates and isotropic or equivalent isotropic displacement parameters (Å^2^)

**Table:**

	*x*	*y*	*z*	*U*_iso_*/*U*_eq_	Occ. (<1)
Zn1	0.62107 (4)	0.44256 (4)	0.58271 (3)	0.05155 (18)	
Cl1	0.47634 (10)	0.38528 (8)	0.44263 (7)	0.0603 (3)	
O10	0.6920 (3)	0.2883 (2)	0.5950 (2)	0.0606 (7)	
H10	0.6421	0.2376	0.5639	0.073*	
N1	0.7553 (3)	0.4677 (3)	0.7236 (2)	0.0572 (9)	
C10	0.8077 (4)	0.3716 (4)	0.7702 (3)	0.0635 (12)	
H10A	0.7484	0.3298	0.7810	0.076*	
H10B	0.8612	0.3875	0.8319	0.076*	
C11	0.8689 (4)	0.3121 (3)	0.7133 (3)	0.0576 (11)	
C12	0.8065 (4)	0.2698 (3)	0.6261 (3)	0.0531 (10)	
C13	0.8592 (4)	0.2119 (3)	0.5741 (3)	0.0645 (12)	
H13	0.8176	0.1860	0.5152	0.077*	
C14	0.9746 (5)	0.1929 (4)	0.6105 (4)	0.0788 (15)	
H14	1.0102	0.1532	0.5761	0.095*	
C15	1.0369 (5)	0.2315 (4)	0.6964 (4)	0.0830 (16)	
H15	1.1143	0.2177	0.7205	0.100*	
C16	0.9841 (4)	0.2916 (4)	0.7478 (4)	0.0757 (14)	
H16	1.0269	0.3184	0.8060	0.091*	
C20	0.6837 (4)	0.5105 (4)	0.7793 (3)	0.0656 (12)	
H20A	0.6611	0.5785	0.7579	0.079*	
H20B	0.7276	0.5133	0.8462	0.079*	
C21	0.5802 (4)	0.4470 (3)	0.7672 (3)	0.0613 (11)	
N22	0.5338 (3)	0.4064 (3)	0.6806 (2)	0.0550 (9)	
C23	0.4413 (4)	0.3488 (4)	0.6644 (4)	0.0654 (12)	
H23	0.4105	0.3197	0.6046	0.078*	
C24	0.3901 (5)	0.3313 (4)	0.7336 (4)	0.0838 (16)	
H24	0.3257	0.2908	0.7210	0.101*	
C25	0.4356 (6)	0.3747 (5)	0.8221 (4)	0.0969 (19)	
H25	0.4010	0.3657	0.8693	0.116*	
C26	0.5320 (5)	0.4309 (4)	0.8391 (4)	0.0836 (16)	
H26	0.5654	0.4585	0.8992	0.100*	
C29	0.8489 (4)	0.5393 (4)	0.7227 (4)	0.0754 (14)	
H29A	0.9141	0.5008	0.7187	0.090*	
H29B	0.8717	0.5761	0.7824	0.090*	
C30	0.8161 (5)	0.6143 (4)	0.6411 (4)	0.0844 (16)	
H30A	0.7421	0.6425	0.6365	0.101*	
H30B	0.8708	0.6690	0.6547	0.101*	
C31	0.8117 (5)	0.5679 (4)	0.5478 (4)	0.0785 (15)	
N32	0.7396 (3)	0.4907 (3)	0.5160 (3)	0.0622 (9)	
C33	0.7388 (5)	0.4462 (4)	0.4339 (4)	0.0786 (15)	
H33	0.6886	0.3930	0.4117	0.094*	
C34	0.8076 (7)	0.4745 (6)	0.3813 (5)	0.110 (2)	
H34	0.8059	0.4407	0.3256	0.132*	
C35	0.8784 (7)	0.5538 (7)	0.4133 (7)	0.131 (3)	
H35	0.9246	0.5761	0.3779	0.157*	
C36	0.8832 (5)	0.6015 (5)	0.4963 (6)	0.105 (2)	
H36	0.9328	0.6551	0.5183	0.126*	
O1W	0.5727 (3)	0.1334 (3)	0.5140 (3)	0.1020 (13)	
H1WA	0.5020	0.1486	0.4914	0.122*	
H1WB	0.5999	0.1120	0.4716	0.122*	
Cl2	0.30150 (12)	0.07028 (13)	0.58931 (11)	0.0868 (4)	
O1P	0.2155 (15)	0.0026 (14)	0.5614 (12)	0.141 (11)	0.345 (18)
O1P'	0.2667 (19)	−0.0214 (9)	0.5796 (12)	0.312 (16)	0.655 (18)
O2P	0.3503 (13)	0.0570 (12)	0.5146 (9)	0.094 (6)	0.345 (18)
O2P'	0.3620 (13)	0.0972 (14)	0.5372 (9)	0.280 (13)	0.655 (18)
O3P	0.3813 (14)	0.0494 (19)	0.6654 (11)	0.32 (3)	0.345 (18)
O3P'	0.3603 (13)	0.0915 (9)	0.6789 (5)	0.193 (7)	0.655 (18)
O4P	0.2611 (19)	0.1602 (10)	0.5814 (18)	0.37 (4)	0.345 (18)
O4P'	0.2149 (12)	0.1330 (15)	0.5729 (12)	0.333 (16)	0.655 (18)

### Atomic displacement parameters (Å^2^)

**Table:**

	*U*^11^	*U*^22^	*U*^33^	*U*^12^	*U*^13^	*U*^23^
Zn1	0.0624 (3)	0.0507 (3)	0.0425 (3)	0.0015 (2)	0.0173 (2)	−0.0019 (2)
Cl1	0.0718 (7)	0.0522 (6)	0.0512 (6)	0.0066 (5)	0.0105 (5)	−0.0085 (5)
O10	0.0609 (19)	0.0487 (16)	0.0676 (19)	0.0033 (14)	0.0125 (15)	−0.0097 (14)
N1	0.065 (2)	0.055 (2)	0.0480 (19)	−0.0006 (17)	0.0122 (16)	−0.0086 (16)
C10	0.069 (3)	0.074 (3)	0.044 (2)	0.008 (2)	0.012 (2)	0.005 (2)
C11	0.063 (3)	0.056 (3)	0.054 (2)	0.009 (2)	0.019 (2)	0.012 (2)
C12	0.063 (3)	0.042 (2)	0.058 (2)	0.005 (2)	0.025 (2)	0.0069 (19)
C13	0.076 (3)	0.054 (3)	0.070 (3)	0.005 (2)	0.032 (3)	0.002 (2)
C14	0.088 (4)	0.068 (3)	0.096 (4)	0.011 (3)	0.052 (3)	0.009 (3)
C15	0.060 (3)	0.090 (4)	0.105 (4)	0.013 (3)	0.034 (3)	0.023 (3)
C16	0.068 (3)	0.089 (4)	0.066 (3)	0.003 (3)	0.015 (3)	0.012 (3)
C20	0.085 (3)	0.066 (3)	0.043 (2)	0.005 (3)	0.015 (2)	−0.013 (2)
C21	0.080 (3)	0.056 (3)	0.051 (2)	0.016 (2)	0.024 (2)	0.000 (2)
N22	0.064 (2)	0.055 (2)	0.050 (2)	0.0066 (18)	0.0238 (17)	0.0015 (16)
C23	0.074 (3)	0.059 (3)	0.071 (3)	0.007 (2)	0.032 (3)	0.007 (2)
C24	0.086 (4)	0.075 (4)	0.105 (4)	0.003 (3)	0.051 (3)	0.015 (3)
C25	0.124 (5)	0.102 (5)	0.090 (4)	0.007 (4)	0.070 (4)	0.012 (4)
C26	0.115 (5)	0.087 (4)	0.061 (3)	0.018 (4)	0.044 (3)	0.002 (3)
C29	0.080 (3)	0.069 (3)	0.073 (3)	−0.014 (3)	0.017 (3)	−0.014 (3)
C30	0.072 (3)	0.061 (3)	0.120 (5)	−0.008 (3)	0.027 (3)	−0.005 (3)
C31	0.079 (3)	0.060 (3)	0.105 (4)	0.013 (3)	0.042 (3)	0.015 (3)
N32	0.073 (2)	0.053 (2)	0.068 (2)	0.003 (2)	0.032 (2)	0.0069 (19)
C33	0.089 (4)	0.090 (4)	0.068 (3)	0.025 (3)	0.041 (3)	0.019 (3)
C34	0.136 (6)	0.121 (6)	0.102 (5)	0.042 (5)	0.077 (5)	0.039 (4)
C35	0.133 (7)	0.137 (7)	0.162 (8)	0.043 (6)	0.103 (6)	0.068 (6)
C36	0.089 (4)	0.089 (4)	0.156 (6)	0.003 (3)	0.065 (5)	0.036 (5)
O1W	0.081 (2)	0.078 (3)	0.142 (4)	−0.003 (2)	0.025 (2)	−0.037 (2)
Cl2	0.0735 (9)	0.1060 (12)	0.0876 (9)	−0.0081 (8)	0.0345 (8)	−0.0347 (8)
O1P	0.147 (14)	0.137 (18)	0.108 (13)	−0.099 (13)	−0.005 (12)	0.025 (13)
O1P'	0.50 (4)	0.166 (13)	0.37 (3)	−0.155 (18)	0.28 (3)	−0.142 (17)
O2P	0.122 (11)	0.103 (10)	0.066 (7)	−0.021 (9)	0.041 (7)	−0.039 (7)
O2P'	0.315 (19)	0.41 (3)	0.185 (13)	−0.25 (2)	0.177 (14)	−0.159 (15)
O3P	0.107 (16)	0.50 (7)	0.27 (4)	0.09 (3)	−0.074 (19)	0.14 (4)
O3P'	0.36 (2)	0.140 (9)	0.064 (5)	−0.051 (10)	0.037 (8)	−0.027 (5)
O4P	0.105 (18)	0.127 (18)	0.84 (10)	0.018 (13)	0.07 (3)	−0.24 (4)
O4P'	0.092 (9)	0.48 (4)	0.37 (2)	0.118 (15)	−0.027 (10)	−0.17 (2)

### Geometric parameters (Å, °)

**Table:**

Zn1—N32	2.096 (4)	C23—H23	0.9300
Zn1—N22	2.103 (3)	C24—C25	1.380 (8)
Zn1—O10	2.212 (3)	C24—H24	0.9300
Zn1—N1	2.252 (3)	C25—C26	1.360 (8)
Zn1—Cl1	2.4048 (12)	C25—H25	0.9300
Zn1—Cl1^i^	2.5555 (12)	C26—H26	0.9300
Cl1—Zn1^i^	2.5555 (12)	C29—C30	1.519 (7)
O10—C12	1.368 (5)	C29—H29A	0.9700
O10—H10	0.9333	C29—H29B	0.9700
N1—C20	1.491 (5)	C30—C31	1.494 (8)
N1—C10	1.498 (6)	C30—H30A	0.9700
N1—C29	1.499 (6)	C30—H30B	0.9700
C10—C11	1.512 (6)	C31—N32	1.345 (6)
C10—H10A	0.9700	C31—C36	1.400 (8)
C10—H10B	0.9700	N32—C33	1.344 (6)
C11—C16	1.383 (6)	C33—C34	1.366 (8)
C11—C12	1.400 (6)	C33—H33	0.9300
C12—C13	1.380 (6)	C34—C35	1.358 (11)
C13—C14	1.383 (7)	C34—H34	0.9300
C13—H13	0.9300	C35—C36	1.364 (10)
C14—C15	1.365 (8)	C35—H35	0.9300
C14—H14	0.9300	C36—H36	0.9300
C15—C16	1.391 (7)	O1W—H1WA	0.8581
C15—H15	0.9300	O1W—H1WB	0.8444
C16—H16	0.9300	Cl2—O2P'	1.274 (9)
C20—C21	1.494 (7)	Cl2—O3P	1.277 (11)
C20—H20A	0.9700	Cl2—O1P'	1.283 (10)
C20—H20B	0.9700	Cl2—O4P	1.284 (11)
C21—N22	1.344 (5)	Cl2—O4P'	1.318 (10)
C21—C26	1.382 (6)	Cl2—O3P'	1.329 (8)
N22—C23	1.334 (6)	Cl2—O1P	1.354 (10)
C23—C24	1.374 (6)	Cl2—O2P	1.418 (10)
			
N32—Zn1—N22	165.61 (15)	C23—N22—C21	119.6 (4)
N32—Zn1—O10	90.28 (13)	C23—N22—Zn1	126.7 (3)
N22—Zn1—O10	90.33 (12)	C21—N22—Zn1	113.7 (3)
N32—Zn1—N1	88.07 (14)	N22—C23—C24	121.7 (5)
N22—Zn1—N1	77.69 (14)	N22—C23—H23	119.1
O10—Zn1—N1	84.28 (12)	C24—C23—H23	119.1
N32—Zn1—Cl1	97.72 (11)	C23—C24—C25	119.0 (5)
N22—Zn1—Cl1	96.67 (11)	C23—C24—H24	120.5
O10—Zn1—Cl1	87.09 (8)	C25—C24—H24	120.5
N1—Zn1—Cl1	169.64 (10)	C26—C25—C24	119.0 (5)
N32—Zn1—Cl1^i^	91.34 (10)	C26—C25—H25	120.5
N22—Zn1—Cl1^i^	89.29 (10)	C24—C25—H25	120.5
O10—Zn1—Cl1^i^	174.95 (8)	C25—C26—C21	120.0 (5)
N1—Zn1—Cl1^i^	100.55 (9)	C25—C26—H26	120.0
Cl1—Zn1—Cl1^i^	87.95 (4)	C21—C26—H26	120.0
Zn1—Cl1—Zn1^i^	92.05 (4)	N1—C29—C30	113.7 (4)
C12—O10—Zn1	122.4 (2)	N1—C29—H29A	108.8
C12—O10—H10	119.6	C30—C29—H29A	108.8
Zn1—O10—H10	116.2	N1—C29—H29B	108.8
C20—N1—C10	108.4 (3)	C30—C29—H29B	108.8
C20—N1—C29	110.6 (4)	H29A—C29—H29B	107.7
C10—N1—C29	108.5 (4)	C31—C30—C29	112.7 (4)
C20—N1—Zn1	100.0 (2)	C31—C30—H30A	109.0
C10—N1—Zn1	113.0 (3)	C29—C30—H30A	109.0
C29—N1—Zn1	116.0 (3)	C31—C30—H30B	109.0
N1—C10—C11	114.1 (3)	C29—C30—H30B	109.0
N1—C10—H10A	108.7	H30A—C30—H30B	107.8
C11—C10—H10A	108.7	N32—C31—C36	120.9 (6)
N1—C10—H10B	108.7	N32—C31—C30	118.0 (4)
C11—C10—H10B	108.7	C36—C31—C30	121.2 (6)
H10A—C10—H10B	107.6	C33—N32—C31	118.1 (4)
C16—C11—C12	118.3 (4)	C33—N32—Zn1	118.4 (3)
C16—C11—C10	122.0 (4)	C31—N32—Zn1	123.4 (3)
C12—C11—C10	119.5 (4)	N32—C33—C34	123.8 (6)
O10—C12—C13	121.8 (4)	N32—C33—H33	118.1
O10—C12—C11	117.3 (4)	C34—C33—H33	118.1
C13—C12—C11	120.9 (4)	C35—C34—C33	117.5 (7)
C12—C13—C14	119.3 (5)	C35—C34—H34	121.3
C12—C13—H13	120.3	C33—C34—H34	121.3
C14—C13—H13	120.3	C34—C35—C36	121.2 (7)
C15—C14—C13	120.8 (5)	C34—C35—H35	119.4
C15—C14—H14	119.6	C36—C35—H35	119.4
C13—C14—H14	119.6	C35—C36—C31	118.5 (7)
C14—C15—C16	119.8 (5)	C35—C36—H36	120.7
C14—C15—H15	120.1	C31—C36—H36	120.7
C16—C15—H15	120.1	H1WA—O1W—H1WB	112.3
C11—C16—C15	120.8 (5)	O2P'—Cl2—O1P'	115.8 (9)
C11—C16—H16	119.6	O3P—Cl2—O4P	117.1 (10)
C15—C16—H16	119.6	O2P'—Cl2—O4P'	107.6 (9)
N1—C20—C21	110.4 (3)	O1P'—Cl2—O4P'	110.7 (7)
N1—C20—H20A	109.6	O2P'—Cl2—O3P'	107.3 (7)
C21—C20—H20A	109.6	O1P'—Cl2—O3P'	111.9 (8)
N1—C20—H20B	109.6	O4P'—Cl2—O3P'	102.4 (7)
C21—C20—H20B	109.6	O3P—Cl2—O1P	116.5 (11)
H20A—C20—H20B	108.1	O4P—Cl2—O1P	110.0 (8)
N22—C21—C26	120.6 (5)	O3P—Cl2—O2P	105.0 (9)
N22—C21—C20	116.1 (4)	O4P—Cl2—O2P	106.8 (11)
C26—C21—C20	123.2 (4)	O1P—Cl2—O2P	99.2 (8)
			
N32—Zn1—Cl1—Zn1^i^	−91.08 (11)	C20—C21—N22—C23	179.9 (4)
N22—Zn1—Cl1—Zn1^i^	89.05 (10)	C26—C21—N22—Zn1	179.4 (4)
O10—Zn1—Cl1—Zn1^i^	179.03 (8)	C20—C21—N22—Zn1	0.4 (5)
N1—Zn1—Cl1—Zn1^i^	145.4 (5)	N32—Zn1—N22—C23	−166.2 (5)
N32—Zn1—O10—C12	45.9 (3)	O10—Zn1—N22—C23	−73.8 (4)
N22—Zn1—O10—C12	−119.7 (3)	N1—Zn1—N22—C23	−157.9 (4)
N1—Zn1—O10—C12	−42.1 (3)	Cl1—Zn1—N22—C23	13.3 (4)
Cl1—Zn1—O10—C12	143.6 (3)	Cl1^i^—Zn1—N22—C23	101.2 (4)
N32—Zn1—N1—C20	141.0 (3)	N32—Zn1—N22—C21	13.3 (7)
N22—Zn1—N1—C20	−37.0 (3)	O10—Zn1—N22—C21	105.7 (3)
O10—Zn1—N1—C20	−128.6 (3)	N1—Zn1—N22—C21	21.6 (3)
Cl1—Zn1—N1—C20	−94.8 (6)	Cl1—Zn1—N22—C21	−167.2 (3)
Cl1^i^—Zn1—N1—C20	50.0 (3)	Cl1^i^—Zn1—N22—C21	−79.3 (3)
N32—Zn1—N1—C10	−104.0 (3)	C21—N22—C23—C24	1.5 (7)
N22—Zn1—N1—C10	78.0 (3)	Zn1—N22—C23—C24	−179.1 (4)
O10—Zn1—N1—C10	−13.5 (3)	N22—C23—C24—C25	0.1 (8)
Cl1—Zn1—N1—C10	20.2 (7)	C23—C24—C25—C26	−2.1 (9)
Cl1^i^—Zn1—N1—C10	165.0 (3)	C24—C25—C26—C21	2.5 (9)
N32—Zn1—N1—C29	22.2 (3)	N22—C21—C26—C25	−1.0 (8)
N22—Zn1—N1—C29	−155.8 (3)	C20—C21—C26—C25	178.0 (5)
O10—Zn1—N1—C29	112.6 (3)	C20—N1—C29—C30	−89.1 (5)
Cl1—Zn1—N1—C29	146.4 (5)	C10—N1—C29—C30	152.1 (4)
Cl1^i^—Zn1—N1—C29	−68.8 (3)	Zn1—N1—C29—C30	23.7 (5)
C20—N1—C10—C11	171.6 (4)	N1—C29—C30—C31	−74.9 (6)
C29—N1—C10—C11	−68.3 (5)	C29—C30—C31—N32	59.3 (6)
Zn1—N1—C10—C11	61.8 (4)	C29—C30—C31—C36	−118.6 (6)
N1—C10—C11—C16	118.6 (5)	C36—C31—N32—C33	0.6 (7)
N1—C10—C11—C12	−66.2 (5)	C30—C31—N32—C33	−177.3 (4)
Zn1—O10—C12—C13	−126.7 (4)	C36—C31—N32—Zn1	−175.2 (4)
Zn1—O10—C12—C11	53.2 (4)	C30—C31—N32—Zn1	6.9 (6)
C16—C11—C12—O10	177.9 (4)	N22—Zn1—N32—C33	152.3 (5)
C10—C11—C12—O10	2.5 (6)	O10—Zn1—N32—C33	59.9 (3)
C16—C11—C12—C13	−2.2 (6)	N1—Zn1—N32—C33	144.1 (3)
C10—C11—C12—C13	−177.6 (4)	Cl1—Zn1—N32—C33	−27.2 (3)
O10—C12—C13—C14	−177.9 (4)	Cl1^i^—Zn1—N32—C33	−115.3 (3)
C11—C12—C13—C14	2.2 (6)	N22—Zn1—N32—C31	−32.0 (8)
C12—C13—C14—C15	−0.8 (7)	O10—Zn1—N32—C31	−124.4 (4)
C13—C14—C15—C16	−0.6 (8)	N1—Zn1—N32—C31	−40.1 (4)
C12—C11—C16—C15	0.8 (7)	Cl1—Zn1—N32—C31	148.5 (4)
C10—C11—C16—C15	176.1 (5)	Cl1^i^—Zn1—N32—C31	60.4 (4)
C14—C15—C16—C11	0.6 (8)	C31—N32—C33—C34	0.4 (7)
C10—N1—C20—C21	−70.2 (4)	Zn1—N32—C33—C34	176.4 (4)
C29—N1—C20—C21	171.0 (4)	N32—C33—C34—C35	−1.6 (9)
Zn1—N1—C20—C21	48.2 (4)	C33—C34—C35—C36	1.9 (11)
N1—C20—C21—N22	−36.3 (5)	C34—C35—C36—C31	−1.0 (11)
N1—C20—C21—C26	144.7 (4)	N32—C31—C36—C35	−0.3 (9)
C26—C21—N22—C23	−1.0 (6)	C30—C31—C36—C35	177.6 (6)

Symmetry codes: (i) −*x*+1, −*y*+1, −*z*+1.

### Hydrogen-bond geometry (Å, °)

**Table:**

Cg is the centroid of the C11–C16 ring.

**Table:**

*D*—H···*A*	*D*—H	H···*A*	*D*···*A*	*D*—H···*A*
O10—H10···O1W	0.93	1.67	2.598 (4)	170
O1W—H1WA···O2P	0.86	2.34	2.928 (15)	126
O1W—H1WA···O2P'	0.86	2.15	2.766 (13)	128
O1W—H1WB···O1P'^ii^	0.84	2.34	3.117 (19)	153
O1W—H1WB···O2P^ii^	0.84	2.32	2.777 (15)	114
C35—H35···Cg^iii^	0.93	3.14	3.944	148

Symmetry codes: (ii) −*x*+1, −*y*, −*z*+1; (iii) −*x*+2, −*y*+1, −*z*+1.
